# Supplementary material for: Cry4Aa and Cry4Ba Mosquito-Active Toxins Utilize Different Domains in Binding to a Particular Culex ALP Isoform: A Functional Toxin Receptor Implicating Differential Actions on Target Larvae
Source: Toxins (Basel). 2022 Sep 21;14(10):652. doi: 10.3390/toxins14100652 (PMC9607545; doi:10.3390/toxins14100652)
Supplement: Supplementary file 1 [file toxins-14-00652-s001.zip › toxins-1895913-supplementary.pdf]

toxins

Article

Cry4Aa and Cry4Ba Mosquito-Active Toxins Utilize Different Domains in Binding to a Particular *Culex* ALP Isoform: A Functional Toxin Receptor Implicating Differential Actions on Target Larvae

Manussawee Dechkla [1,*], Sathapat Charoenjotivadhanakul [2], Chompounoot Imtong [3], Sarinporn Visitsattapongse [4], Hui-Chun Li [5] and Chanan Angsuthanasombat [2,3,5,*]

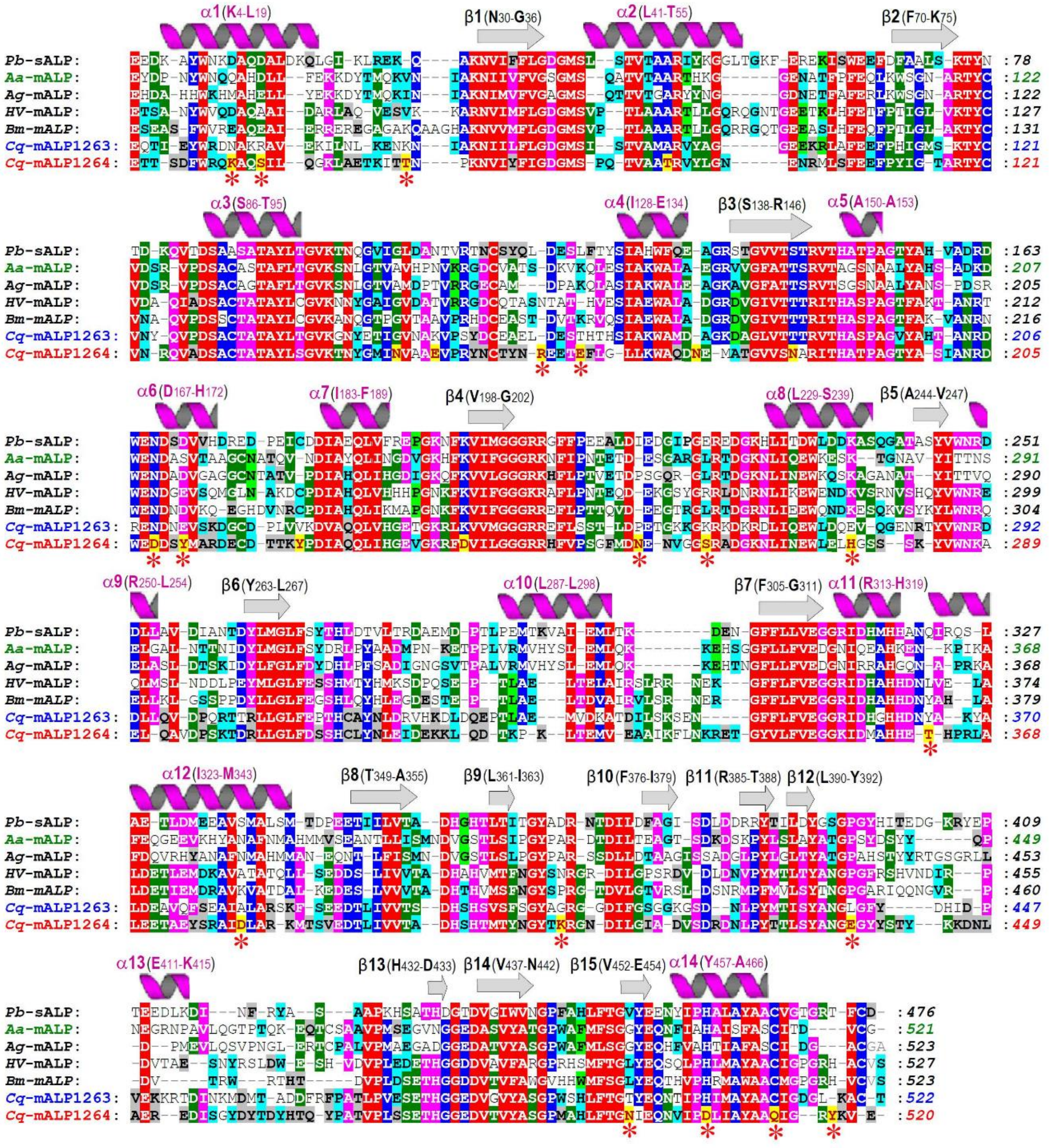

Figure S1. Multiple sequence alignments of Alps. The deduced amino acid sequences of Cq-mALP1263 and Cq-mALP1264 were compared with ALPs from other organisms including the sequences of known 3D-structure shrimp-ALP (Pb-sALP), and that of undetermined structures from other insect species (Aa-mALP, Ag-mALP, Hv-ALP and Bm-ALP). Shrimp-ALP was used as a reference sequence for secondary structures which are shown on top of the corresponding sequence. Amino acids are shaded red, blue, pink and green or cyan to donate degree of homology (7/7), (6/7), (5/7), (4/7) and (3/11), respectively. Predicted surface residues which are unique for Cq- mALP1264 are shown in red and indicated with *.

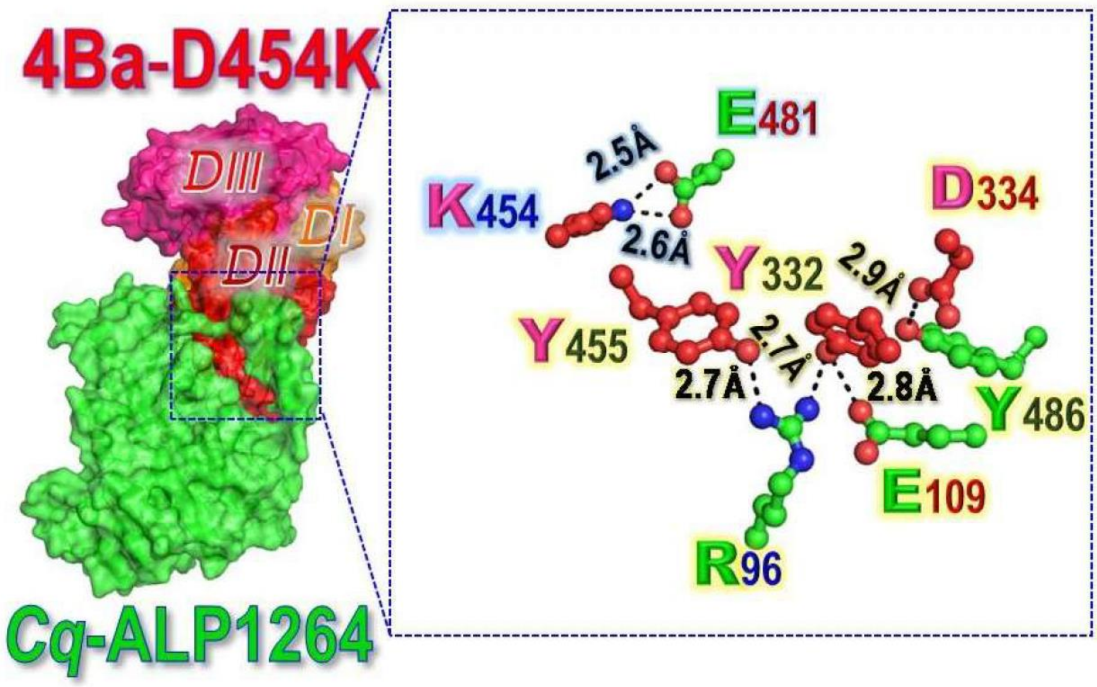

Figure S2. Surface representation of the Cry4Ba-D454K modeled structure interacting with its functional receptor—Cq-mALP1264. *Inset*, zoomed in view of potential receptor-binding residues within DII, i.e. the mutated residue—Lys454 along with other residues in β2-β3 (Tyr332 and Asp334) and β10-β11 (Tyr455) loops, which are represented as ball-and-stick models along with their interacting partner residues.
